# Microscopy Methods for Biofilm Imaging: Focus on SEM and VP-SEM Pros and Cons

**DOI:** 10.3390/biology10010051

**Published:** 2021-01-12

**Authors:** Michela Relucenti, Giuseppe Familiari, Orlando Donfrancesco, Maurizio Taurino, Xiaobo Li, Rui Chen, Marco Artini, Rosanna Papa, Laura Selan

**Affiliations:** 1 Department of Anatomy, Histology, Forensic Medicine and Orthopedics, Sapienza University of Rome, Via Alfonso Borelli 50, 00161 Rome, Italy; giuseppe.familiari@uniroma1.it (G.F.); orlando.donfrancesco@uniroma1.it (O.D.); 2 Department of Clinical and Molecular Medicine, Unit of Vascular Surgery, Sant’Andrea Hospital, Sapienza University of Rome, Via di Grottarossa 1039, 00189 Rome, Italy; maurizio.taurino@uniroma1.it; 3 Key Laboratory of Environmental Medicine Engineering, Ministry of Education, School of Public Health, Southeast University, Nanjing 210096, China; 101011116@seu.edu.cn (X.L.); 101011816@seu.edu.cn (R.C.); 4 Department of Public Health and Infectious Diseases, Sapienza University of Rome, P.le Aldo Moro 5, 00185 Rome, Italy; marco.artini@uniroma1.it (M.A.); rosanna.papa@uniroma1.it (R.P.); laura.selan@uniroma1.it (L.S.)

**Keywords:** scanning electron microscopy, variable pressure scanning electron microscopy, biofilm

## Abstract

**Simple Summary:**

Bacterial biofilms cause infections that are often resistant to antibiotic treatments. Research about the formation and elimination of biofilms cannot be undertaken without detailed imaging techniques. In this review, traditional and cutting-edge microscopy methods to study biofilm structure, ultrastructure, and 3-D architecture, with particular emphasis on conventional scanning electron microscopy and variable pressure scanning electron microscopy, are addressed, with the respective advantages and disadvantages. When ultrastructural characterization of biofilm matrix and its embedded bacterial cells is needed, as in studies on the effects of drug treatments on biofilm, scanning electron microscopy with customized protocols such as the osmium tetroxide (OsO_4_), ruthenium red (RR), tannic acid (TA), and ionic liquid (IL) must be preferred over other methods for the following: unparalleled image quality, magnification and resolution, minimal sample loss, and actual sample structure preservation. The first step to make a morphological assessment of the effect of the various pharmacological treatments on clinical biofilms is the production of images that faithfully reflect the structure of the sample. The extraction of quantitative parameters from images, possible using specific software, will allow for the scanning electron microscopy morphological evaluation to no longer be considered as an accessory technique, but a quantitative method to all effects.

**Abstract:**

Several imaging methodologies have been used in biofilm studies, contributing to deepening the knowledge on their structure. This review illustrates the most widely used microscopy techniques in biofilm investigations, focusing on traditional and innovative scanning electron microscopy techniques such as scanning electron microscopy (SEM), variable pressure SEM (VP-SEM), environmental SEM (ESEM), and the more recent ambiental SEM (ASEM), ending with the cutting edge Cryo-SEM and focused ion beam SEM (FIB SEM), highlighting the pros and cons of several methods with particular emphasis on conventional SEM and VP-SEM. As each technique has its own advantages and disadvantages, the choice of the most appropriate method must be done carefully, based on the specific aim of the study. The evaluation of the drug effects on biofilm requires imaging methods that show the most detailed ultrastructural features of the biofilm. In this kind of research, the use of scanning electron microscopy with customized protocols such as osmium tetroxide (OsO_4_), ruthenium red (RR), tannic acid (TA) staining, and ionic liquid (IL) treatment is unrivalled for its image quality, magnification, resolution, minimal sample loss, and actual sample structure preservation. The combined use of innovative SEM protocols and 3-D image analysis software will allow for quantitative data from SEM images to be extracted; in this way, data from images of samples that have undergone different antibiofilm treatments can be compared.

## 1. Introduction

Surface-attached microbial agglomerations were for the first time named as a “biofilm” by William J. Costerton in 1978 [1]. In the following years, he perfected this definition by also considering the host role and the three-dimensional (3-D) architecture. The definition of biofilm was thus implemented, expanding the concept toward a complex community of microorganisms living attached to a surface or interface, being enclosed in an exopolysaccharide matrix (Eps) of microbial and host origin arranged in a three-dimensional (3-D) architecture [2]. Bacterial species in biofilms exhibit cooperation [3], behaving as complex multi-cellular organisms [4]. Eps composition is complex and it may contain polysaccharides, proteins, nucleic acid, lipids, and metals. [5]. The complex array of chemically and functionally diverse biomolecules in the Eps has been termed the matrixome [6], which contributes to the peculiar characteristics of biofilm behavior. According to the National Institutes of Health (NIH), bacterial biofilms are responsible for up to 75% of infectious diseases in humans, as implant-related infections and/or tissue-associated infections [7]. In the European Union and European Economic Area, 8.9 million healthcare-associated infection episodes per year are due to biofilms [8]. These infections are often recurrent and resistant to antibiotic treatments [9,10] due to the particular characteristics of Eps that protect the resident microorganisms from the effects of host immunity or administered antimicrobial drugs [11]. It is of crucial importance nowadays to design or screen anti-biofilm molecules that can effectively minimize and eradicate biofilm-related infections. In this kind of investigation, the use of different microscopy techniques is required to better understand the intimate details of the biofilms’ ultrastructure, their 3-D organization, cell population behavior, and reactions after drug treatments [12]. The development of novel morphological investigation approaches is therefore crucial.

## 2. Microscopy Techniques Applied to Biofilm Imaging

### 2.1. Light Microscopy (LM)

Light microscopy (LM) is a basic imaging technique that is useful for providing the visual identification of biofilm presence and also has significant prognostic value [13]. It can be used for quantitative assessment of biofilm biomass, being easy and low cost to perform [14,15]. However, light microscopy has limited magnification and resolution, so it cannot be applied to describe the finest details of biofilm cell morphology or Eps architecture, but it can be coupled with Scanning electron microscopy (SEM) and Transmission electron microscopy (TEM) in correlative studies as in [16]. In this study on teeth microflora, light microscopy observation of semi-thin sections from demineralized teeth provided the best overall perspective of the root canal, enabling larger areas to be observed at low magnification. Samples observed with SEM did not show bacteria in dentine tubules, in contrast, when the same samples were demineralized and included in resin, their semi-thin section LM images revealed the presence of bacteria, then TEM images confirmed the LM findings [16].

### 2.2. Confocal Laser Scanning Microscopy (CLSM)

Confocal laser scanning microscopy (CLSM) allows for the quantitative evaluation of structural parameters as biovolume (cells overall volume in the observation field), thickness, and roughness. Sample 3-D architecture representation and its time-dependent variation (real-time 4-D) can also be achieved [17]. CLSM was used in combination with a fluorescent stain and was successfully applied on different biofilms species [18,19,20,21]. The CLSM resolution level is singe cell dimension and using pathogen-specific probes labeled with different fluorescent dyes (FISH followed by CLSM) as described in [22], identification of a single species in multispecies samples is allowed. With the same approach, interspecies competition assessment as well as interference in-between species were analyzed [23]. In studies assessing drug antimicrobial effects, CLSM was used, together with specific fluorophores, to discriminate between live or dead bacterial cells, localizing also their spatial distribution [24,25,26,27,28]. CLSM is a method of choice for biofilm visualization and quantification. Unfortunately, CLSM biofilm analysis has limitations due to the use of fluorophores, the existence of a limited number of reporter molecules, and the signal of interest might be hidden by the interference of intrinsic biofilm fluorescence with that of the probe.

### 2.3. Atomic Force Microscopy (AFM)

Bacteria respond to different mechanical signals [29] like adhesion forces originating during adhesion processes. During these events, bacterial surfaces deform [30], modifying the intra-bilayer pressure profile [31], which, in turn, changes bacterial gene expressions, transforming a planktonically growing cell into a biofilm growing one. Atomic force microscopy (AFM) allows for the quantification of adhesion forces existing among living cells, and between cells and surfaces [32,33]. The knowledge of how adhesion and viscoelasticity can modulate biofilm development may be important in the design of biofilm control strategies. Viscoelastic properties of biofilms influence antimicrobial penetration and removal of biofilm from surfaces and therefore performs a role in their protection against mechanical and chemical challenges [34]. This approach was recently used to demonstrate how amyloid protein production dramatically increases the stiffness of Pseudomonas biofilms [35]. AFM has been applied to obtain insights into biofilm 3-D developmental patterns [36,37,38,39,40,41]. Atomic force microscopy (AFM) allows for the quantification of biofilm biomass in terms of thickness and Eps amount based on height and roughness analyses from AFM images [42,43,44,45,46,47,48,49]. Vantages, disadvantages and application fields of non-electron microscopic techniques are summarized in Table 1.

## 3. Scanning Electron Microscopy Techniques Applied to Biofilm Study

In studies connecting differences in biofilm composition with function, the visualization of microbial biofilms in their finest details is mandatory, and to accomplish this task, electron microscopy has no rivals. However, to avoid artifact formation, sample preparation protocols, instrument selection, and acquisition parameters must be finely tuned and customized to the specific sample. The following will discuss the electron microscopy techniques that have been used in studying biofilms: conventional SEM, field emission SEM (FESEM), variable pressure SEM (VP-SEM), environmental SEM (ESEM), and the more recent ambiental SEM (ASEM), ending with the cutting edge cryo-SEM and focused ion beam SEM (FIB SEM).

### 3.1. Conventional Scanning Electron Microscope (SEM)

Conventional SEM and FESEM are the best methods for biofilm visualization if high magnification and high-resolution images are needed to accurately describe biofilm morphology [50,51,52]. In comparative analyses, like the evaluation of the anti-biofilm effects of a drug/treatment, it appears as an extremely useful tool [11], since the results of SEM imaging are highly correlated with those from other analytical methods [53,54,55,56,57,58,59]. The use of dedicated SEM imaging software for biofilm image analysis has allowed for the quantitative morphological analysis of biofilm by several authors [56,60,61,62,63]. The undisputed advantages of SEM and FESEM consist of the combined ability to image with a wide range of magnifications (20 to 30,000×) coupled with high resolution (from 50 to 100 nm) and depth of field. Furthermore, 3D image analysis software allows for data extraction and quantification of detailed morphological findings. In biofilm study, however, some inconveniences may occur. Dehydration and coating with a conductive material can cause Eps collapse and overall biofilm shrinkage [64]. During a critical point drying procedure, ethanol flow could cause possible extraction of the sample material [65], so hexamethyldisilazane (HDMS) drying can be used as a valuable, time-saving, and inexpensive option [53,66,67]. Conventional SEM and FESEM limitations can be overcome using highly tailored protocols for biofilm or using alternative SEM modalities such as VP-SEM, cryo-SEM, and environmental-SEM (ESEM).

### 3.2. Variable Pressure Scanning Electron Microscopy (VP-SEM)

High-resolution imaging in challenging experimental conditions is made possible by VP-SEM, as this technique allows for the visualization of fully hydrated and out-gassing samples with little or no sample preparation procedures [68,69,70]. In studies focusing on biofilm properties, the characterization of hydrated biofilm is fundamental, and conventional high-vacuum SEM (requiring dehydration and drying) causes matrix Eps collapse, giving a deformed biofilm appearance and disfigured architecture. VP-SEM, instead, requires no dehydration and drying, and, if coupled with appropriate fixation, followed by heavy metal staining that enhances contrast and resolution, can be used in bacterial [71,72,73,74] and fungal [75] biofilm characterization. The primary and secondary most used fixatives are aldehydes (glutaraldehyde or paraformaldehyde cross-links proteins), osmium tetroxide (OsO4), which binds specifically to lipids [76], allowing for excellent preservation of fine features. It is used both as a fixative both and as a contrasting agent in studies of hydrated fungal biofilms [75] where lipid droplet inclusion is highlighted. Heavy metal staining is a “trusted” method that has been used since the dawn of electron microscopy. Ruthenium Red was recently used to provide contrast in the VP-SEM imaging of bacterial [71] and fungal biofilms [75]. It is a cation, and specifically binds to Eps polyanionic constituents [77,78,79], but also phospholipid membranes and Ca^2+^-binding proteins, and these abilities make it an excellent stain for both biofilm ECM and hyphal components. Ruthenium Red staining can be used in combination with OsO_4_ staining [71,75]. Ruthenium tetroxide (RuO_4_) staining is less toxic than OsO_4_, it binds with greater strength to polar lipids and, at the same time, stains proteins, monosaccharides, and glycogen [80,81]. Recently, it has been used for hydrated specimens in VP-SEM imaging [75]. Other “historical” stains used in transmission electron microscopy and useful in VPSEM are Alcian Blue, Safranin O, L-lysine, [82,83], uranyl acetate, [84,85,86] phosphotungstic acid, and tannic acid [87,88,89].

### 3.3. Comparing Conventional SEM and VP-SEM on S. mutans Samples

Few interesting papers have compared biofilm observation by conventional SEM, VPSEM, and different protocols for each technique. An *S. mutans* biofilm was analyzed by conventional SEM, the SEM protocol with Ruthenium Red, and VP-SEM by [72]. They suggested that VPSEM is the most accurate technique to observe the *S. mutans* biofilms’ Eps matrix topography as the SEM dehydration steps and high vacuum conditions are noxious for Eps integrity, even if RR is used. Different results were obtained in [50], where in this study, the authors observed the *S. mutans* biofilm (*S. mutans* CCUG 35176, obtained from the Culture Collection University of Göteborg, CCUG, grown at 37 °C under aerobic conditions on aluminum discs) by SEM and VP-SEM. The *S. mutans* biofilm was prepared with different protocols: the conventional SEM sample preparation procedure; VP-SEM protocol, and new protocols adopting osmium tetroxide (OsO_4_), ruthenium red (RR), tannic acid (TA) impregnation, and ionic liquid (IL) drop-casting, which replaced the sputter coating procedure and were discussed above for the RR properties. Concerning tannic acid, it reacts with osmium tetroxide and increases lipid retention, forming complexes that link to proteins and carbohydrates [90]. Consequently, their combined use enhanced extracellular matrix resistance to mechanical damage during sample preparation (specimen hardening, [91,92,93,94]). The sample acquires intrinsic conductive properties into the bulk, not only on the surface (as it happens with sputter coating), but enhanced image contrast without charging phenomena, and 3-D observation of sub-surface structures under higher voltages are allowed [95]. At room temperature, ionic liquids are molten salts, they have high electronic conductivity, and extremely low vapor pressure [96,97]. Recently, they have been used in SEM as a substitute for metal coating [98]. An IL covering keeps the biofilm hydrated under high vacuum SEM conditions (IL resists evaporation), so the Eps is preserved, thus avoiding dehydration steps and critical point drying procedures. The protocols used in [50] are summarized in Table 2.

#### 3.3.1. *S. mutans* Observed by Conventional SEM

The conventional SEM protocol consists of (a) fixation in glutaraldehyde 2.5% in PB pH 7.4 and post-fixation in OsO_4_ 1% solution in H2O; (b) dehydration steps in ascending alcohol series; (c) critical point drying by CO_2_ substitution; and (d) platinum sputter coating. According to the specific characteristics of the biological sample to be examined, every single step can be modified [99,100,101]. The typical structure of a biofilm is a delicate three-dimensional network, easily damageable during preparation procedures. To avoid network disruption due to ethanol flow during critical point drying, we dried our samples in ascending hexamethyldisilazane (HDMS) series.

**Figure 1:**
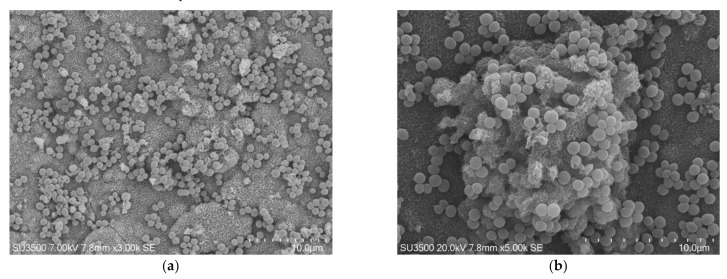
*S. mutans* prepared by conventional scanning electron microscope (SEM) protocol. (**a**) SEM, 3000×. *S. mutans* spherical bacterial cells were scattered on biofilm matrix compact surface. Image was captured from the same sample observed in Figure 1a from [50]; (**b**) SEM, 5000×. Increasing magnification *S. mutans* spherical bacterial cells appeared clustered in small groups on the surface of a rough and dense extracellular matrix (Eps).

**Figure 2:**
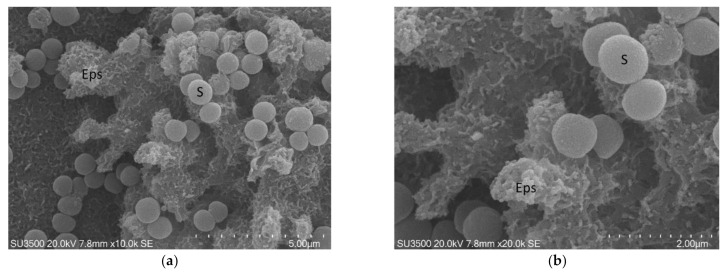
*S. mutans* prepared by conventional SEM procedure. (**a**) SEM, 10,000×. Eps forms a canalicular system of compact trabeculae with spiny surface. Bacterial cells, S, are adherent to the Eps surface. Eps: extracellular polymeric substance. Image was captured from the same sample observed in [50] Figure 1d; (**b**) SEM, 20,000×. Bacterial cells appear irregular, Eps micro-canalicular system is not developed, only superficial holes are visible. Bacterial cells lay down on the Eps surface, and they appear naked, without a matrix covering. Bacterial cells are sometimes fragmented or indented; Eps showed a compact aspect due to the collapse of its fine structure. Bacterial cells, uncovered by the matrix, rest on the Eps surface. Eps: extracellular polymeric substance, S: *S. mutans*. Image was captured from the same sample observed in [50]. Figure 1f.

The images, as shown in Figure 1 and Figure 2, belong to samples prepared with the conventional SEM protocol. These pictures have high resolution, depth of field, and a wide magnification range. The conventional SEM protocol allowed samples to resist for a long time under high vacuum conditions and the action of an electron beam with 20 kV voltage. Carefully observing the images in Figure 2, captured at magnifications 10 k, the downsides of conventional SEM emerge clearly. The shape of the bacterial cells was irregular, and the Eps looked like a dense and shrunken mass with a rough surface. In samples prepared with this protocol, Eps appeared more as a mass dotted with holes rather than a structure within which an intricate and well-developed micro canalicular system developed.

#### 3.3.2. *S. mutans* Observed by VP-SEM

**Figure 3:**
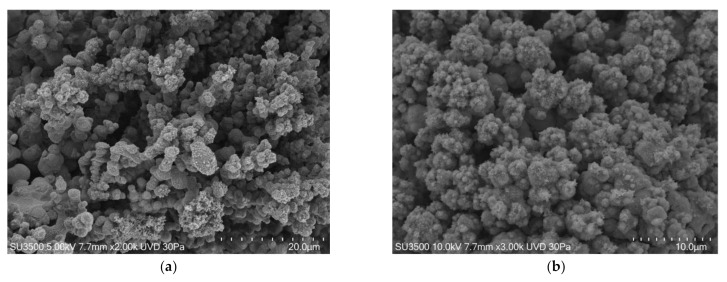
*S. mutans* prepared by conventional VP-SEM procedure. (**a**) VP-SEM 2000×. An intricate micro-canalicular system develops among bacterial towers. Image was captured from the same sample observed in [50] Fig. (**b**) VP-SEM 3000×. Hydration preservation confers biofilm a spongy aspect, without shrinking or any sign of collapse. Image was captured from the same sample observed in [50] Figure 2d.

**Figure 4:**
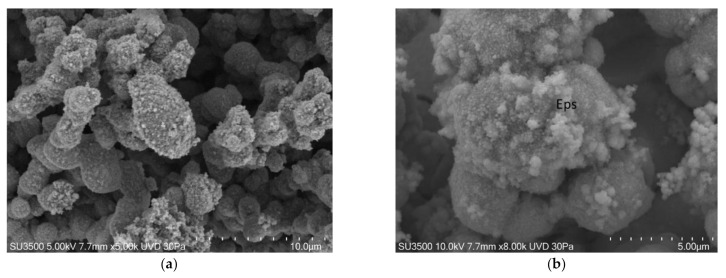
*S. mutans* prepared by conventional variable pressure SEM (VP-SEM) procedure. (**a**) VP-SEM 5000×. Bacterial towers are lined by a superficial granulation representing Eps secretion. Image was captured from the same sample observed in [50] Figure 2f. (**b**) VP-SEM 8000×. On *S. mutans* cells surface is clearly visible the fine graininess of freshly secreted Eps components. Image is the same of [50] Figure 2e.

We can state that using the VP-SEM technique safeguards Eps better than conventional SEM, and the images in Figure 3 clearly show that bacterial towers and the intricate micro-channels system that characterize biofilms had a well-preserved topography. However, Eps appeared in some areas to be compact while in others, it was spongier. Cells were regularly shaped. In Figure 4, the images at higher magnifications showed the fine graininess of freshly secreted Eps components on the bacterial cell surface. Good quality and very informative pictures were obtained up to 10 k, but at higher magnifications, the overall image quality dropped significantly due to signal-to-noise ratio lowering. The resolution of VP-SEM images was lower than the SEM ones, and the sample resistance time at an operating condition of vacuum 30 Pa and electron beam of 5 kV was short (about 1 h before cracking phenomena appearance).

#### 3.3.3. *S. mutans* OsO_4_-RR-TA-IL Procedure Observed by SEM (High Vacuum, High Voltage Conditions)

**Figure 5:**
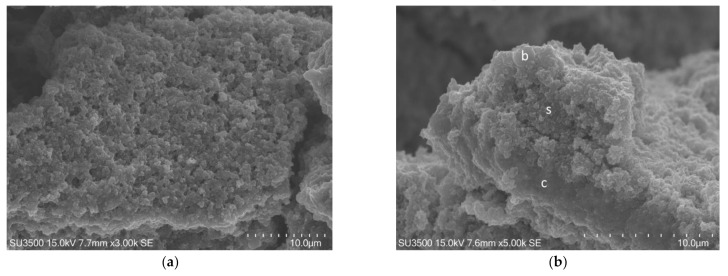
*S. mutans* prepared by the OsO_4_-RR-TA-IL procedure. (**a**) 3000×. The biofilm topography showed a spongy appearance. (**b**) 5000×. The biofilm topography showed both compact, c, and spongy, s, appearance, a single bacterial cell, b, was partially embedded in Eps.

**Figure 6:**
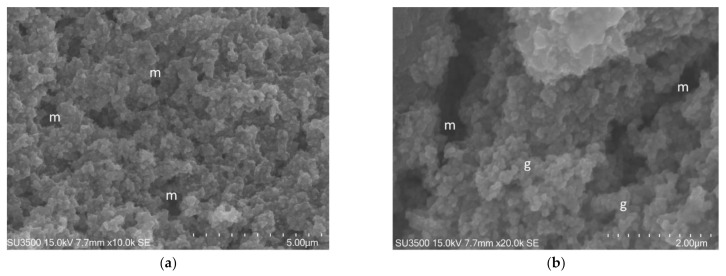
*S. mutans* prepared by the OsO_4_-RR-TA-IL procedure. (**a**) At high magnification, a well-developed micro-canalicular system, m, is visible in the spongy Eps, 10,000×. Image was captured from the same sample observed in Figure 3b from [50]. (**b**). High voltage, high magnification, and high-resolution image of microcanalicular system, m. Fully hydrated Eps appears as a spongy structure formed by globular aggregate, g, at nanometric level, no filaments or collapsed network are visible, 20,000×. Image was captured from the same sample observed in [50] Figure 3f.

**Figure 7:**
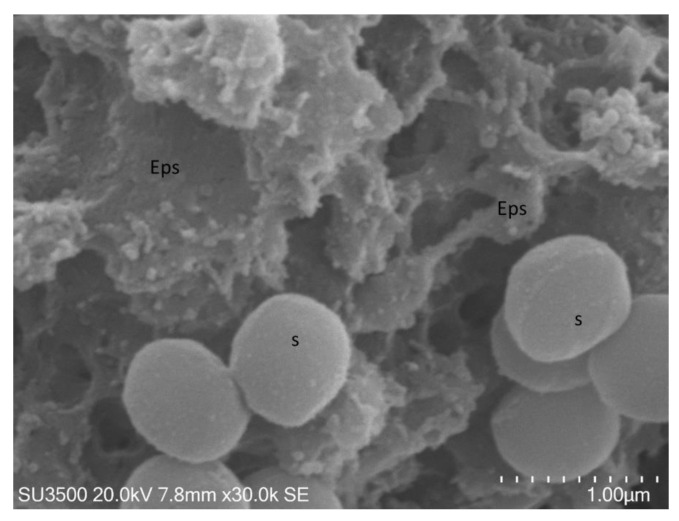
*S. mutans* prepared by the conventional SEM protocol. At high magnification, 30,000×, Eps appeared as a collapsed network of filaments, cell surface, s, was naked, and not covered by Eps.

**Figure 8:**
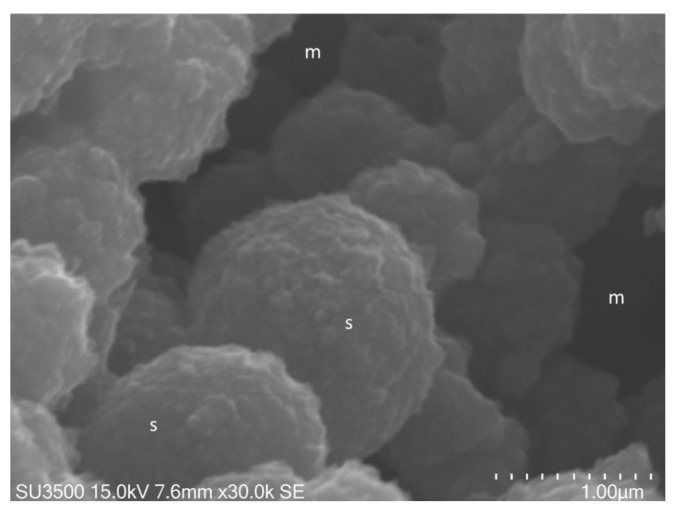
*S. mutans* prepared by the OsO_4_-RR-TA-IL procedure. At high magnification, 30,000×, and high voltage, 15 kV, a globular Eps formed trabeculae of a microcanalicular system, m, and lines the bacterial cells’ surface, s. This high magnification and high-resolution image confirms the value of this protocol in terms of biofilm three-dimensional structure preservation up to the nanometric level.

In studies on the effects of drug treatments on biofilm, ultrastructural characterization of the biofilm matrix and its embedded bacterial cells are needed. In such cases, scanning electron microscopy may be the best choice for its high image quality, magnification, and resolution, but only if protocols are carefully customized to allow imaging of a hydrated sample under high vacuum and high voltage conditions. In the paper [50], an innovative protocol (OsO_4_-RR-TA-IL) was tested. The use of the OsO_4_-RR solution (Table 2) in a post-fixation step was discussed above [78,79,102,103,104]. Tannic acid impregnation was used to harden Eps and to render the sample itself conductive without the appearance of charging phenomena [89,90,91]. Sputter coating allows only the surface to be conductive, so the combined use of OsO_4_-RR in the post-fixation step, followed by tannic acid impregnation, improved observation, allowing visualization of the sub-surface structures like bacterial cell embedded in the biofilm matrix [94]. Finally, to maintain the biofilm in a hydrated state, but to observe it under high vacuum and high voltage conditions, ionic liquid drop-casting was used. In Figure 5 and Figure 6, images of *S. mutans* treated with the OsO_4_-RR-TA-IL procedure are shown. In Figure 7 and Figure 8, images at very high magnification of *S. mutans* prepared with conventional SEM procedure (Figure 7) and OsO4-RR-TA-IL method (Figure 8) are shown. The biofilm surface appeared to be compact in some areas and spongy in others (Figure 5a,b), but the overall topography was well preserved. The biofilm matrix was perforated by a developed and intricate system of microchannels (Figure 6a,b). The biofilm topography was perfectly preserved, and even at the nanometric level, Eps appeared as an intricate 3D network in which no sign of deformation was apparent (Figure 6a,b). The shape of the *S. mutans* cells was smooth and spherical. Bacterial cell emerged from Eps in some areas (Figure 5b), while in others, they were partially embedded in it. In Figure 7 Eps consists in a network of collapsed filaments, in Figure 8 Eps keeps a fine “fluffy cloud” appearance, covering bacterial cell surface. Together with the achievement of high-quality images, the use of the OsO_4_-RR-TA-IL protocol under 30 k in high vacuum conditions allowed a longer observation time than the VP-SEM protocol.

### 3.4. Candida Albicans OsO_4_-RR-TA-IL Procedure Observed at SEM (High Vacuum, High Voltage Conditions)

**Figure 9:**
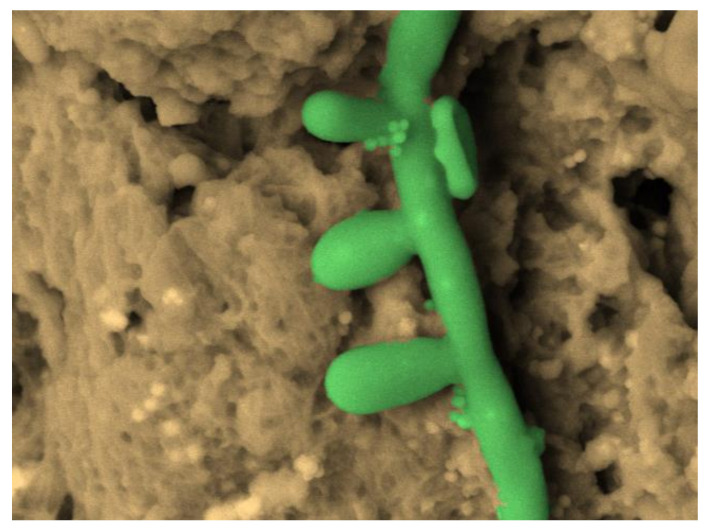
*Candida albicans* hyphae with conidia and spores, SEM OsO_4_-RR-TA-IL protocol 3000×, image artificially colored by software 3D Hitachi Mountains Map (Digital Surf, France).

**Figure 10:**
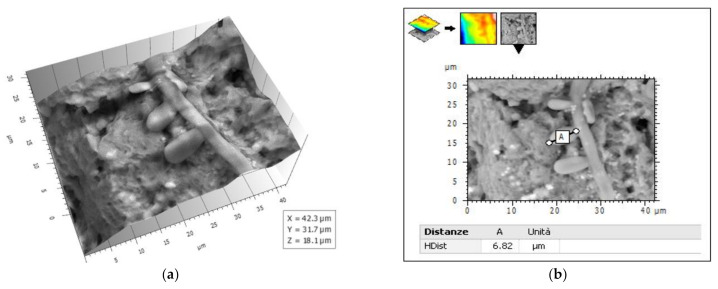
(**a**) 3-D reconstruction from picture in Figure 9 by 3D Hitachi Mountains 7 software. (**b**) Conidium length measure.

Not only bacterial biofilms, but also fungal biofilms, were recently investigated [75] by comparing the SEM, FESEM, and VP-SEM techniques and various experimental producers, also considering Ruthenium Red as an ionic liquid. Results in [75] reached comparable results with those in [50]. They stated that VP-SEM is a superb modality when the visualization of the hydrated 3D biofilm structure is the most important, rather than the finest ultrastructural surface features of single cells (or hyphae in *A. fumigatus*). Thus, when ultrastructural features of cellular and ECM components are desired, high-resolution SEM and FESEM techniques are superior. However, sample preparation parameters should be optimized for any new biofilm sample as the maintenance of the biofilm ultrastructure during SEM analysis is dependent on the sample fixative and fixing time, and drying methods (critical point drying vs. HDMS). In particular, to correctly visualize *A. fumigatus* biofilms by SEM, the authors of [75] suggested using a short primary fixation time (up to 1 h) with a post-fixation with OsO_4_ to enhance the staining of lipids and improve the contrast between cells and ECM. Staining with other heavy-metal reagents such as Ruthenium Red and RuO_4_ may provide more specific contrasting of ECM components. As stated in [50], they also confirmed that sample drying using HDMS was a valuable and rapid alternative to conventional chemical fixation and drying. We tested the OsO_4_-RR-TA-IL protocol on a *Candida albicans* biofilm from an ex vivo sample (unpublished results) and also analyzed the images obtained with a four-quadrant method [100] by Hitachi 3D Mountains Map software (Digital surf, France). The use of this protocol allows for the imaging of the finest details of the *Candida albicans* biofilm, allowing 3D reconstruction and quantification of its actual morphological parameters, measured on the hydrated sample (Figure 9 and Figure 10a,b).

### 3.5. Pros and Cons of Different SEM Protocols

Different SEM protocols can be used to carry out an ultrastructural characterization of a biofilm. To evaluate the advantages and disadvantages of each protocol, the parameters shown in Table 3 were considered, together with the overall informative value given by image magnification and resolution. In our opinion, OsO_4_-RR-TA-IL was revealed as the best protocol for the following reasons: (1) it is a fast procedure (timing comparable with that of VP-SEM protocol, despite having more steps than VP-SEM protocol); (2) modest sample loss during preparation procedures; (3) sample hardening, induced by the conductive staining protocol, increased the resistance of Eps to mechanical damage throughout protocol steps; (4) lacking dehydration and drying steps preserves high Eps water content and Eps actual 3-D structure; (5) high-quality images at magnifications up to 30 K are achieved; and (6) sample resists under operating conditions for a long time (even for 2 h in high vacuum conditions). If an ultrastructural characterization of Eps and the bacterial cells of the biofilm has to be carried out, the OsO_4_-RR-TA-IL protocol has to be preferred over conventional SEM and VP-SEM procedures because it combines the best of both techniques, giving the possibility of achieving images of high quality, at high magnification, and resolution (typical of conventional SEM) in a short time, with a protocol of a few steps, minimal sample loss, and actual sample structure preservation (advantages of VP-SEM).

## 4. Cryo-SEM

Biofilm cryo-fixation is a fast procedure that allows “frozen in time” specimen observation [105]. A frozen biofilm can be freeze-fractured to expose its inner structure, observe bacterial cell, and their interconnections [64,67]. Cryo-SEM can be associated with high-pressure freezing. Combining these two cutting edge techniques for biofilm analysis allows for a detailed visualization of the relationships among the microbial cells and Eps ultrastructure. An innovative method for the preparation of fully hydrated samples of microbial biofilms (*Staphylococcus epidermidis, Candida parapsilosis* and *Candida albicans*) was presented in [106]. Cryo-SEM requires very expensive and highly specialized equipment as well as highly skilled technicians, which is why it has had limited use in biofilm studies. Furthermore, it has lower image resolution compared with conventional SEM and, at high magnifications, the heat generated by the focused electron beam may induce melting and cracking of the frozen surface [64].

## 5. Environmental Scanning Electron Microscopy (ESEM)

Environmental SEM allows for the observation of biofilms without any pretreatment, thus saving their integrity, as they were in the natural state. The sample is directly put into the microscope chamber, whose pressure values are near the environmental value, instead of the very low-pressure values (high vacuum) of a conventional SEM. Hydrated and non-conductive living bacterial biofilms can be visualized, without dehydration artifacts and loss of mass [64,68]. However, the lack of conductivity lowers resolution, and as the sample is wet, a fast image capture setting is required. The sample can easily be damaged under the focused electron beam when a magnification around 10 kX is reached, as a conductive metal coating is absent.

## 6. Atmospheric Scanning Electron Microscopy (ASEM)

Atmospheric scanning electron microscopy (ASEM) allows an inverted SEM to observe a wet sample from below, while an OM simultaneously observes it from above [107]. Biofilms can be cultured, fixed, and imaged in the specialized sample dish (ASEM dish) at atmospheric pressure [108,109]. ASEM, using 30 kV acceleration voltage, allows observation of 2–3 μ m-thick biofilms with 8 nm resolution. An important advantage is that time-consuming sample protocols (usually required for immuno-EM) are not required for immuno-ASEM [108,110]. Additionally, in this technique, heavy metal stains should apply to thick biofilms. In [111], the ability of osmic acid (OA), uranyl acetate (UA), and lead citrate (LC) was tested to stain 24-h biofilms of a clinically isolated strain of methicillin-resistant *S. aureus*. ASEM imaging at 30 kV revealed that sequential staining with OA, UA, and LC drastically improved image contrast. Using this method, biofilm development was visualized over an aqueous environment, revealing that bacterial cells did not align near each other at the bottom of biofilms, but that there were cell-free regions, probably so-called water channels. Thus, imaging of bacterial behavior in multicellular biofilms in liquid state, can be achieved by atmospheric scanning electron microscopy.

## 7. Focused Ion Beam-SEM (FIB-SEM)

Focused ion beam-SEM is a cutting-edge sophisticated technique for the subsurface structure of biofilm imaging. A standard SEM viewing is coupled with FIB milling to obtain 3-D reconstructions by a process termed “slice and view”. The image slices obtained in succession are then stacked by software to reconstruct the 3-D volume [64,112]. Focused-ion-beam (FIB) tomography was used in [112] to study the morphology of early-stage biofilms of *S. aureus* grown on different surfaces to evaluate the role of stress-induced membrane thinning in the planktonic-to-biofilm transition associated with bacterial adhesion. The resolution afforded by FIB allowed changes to be revealed in the cell-envelope thickness, thus relating them with planktonic-to-biofilm transition. Reducing bacterial growth on implant materials is a challenging purpose, and recently, nanostructures that cause contact-dependent cell death by mechanical rupture of bacterial cell membranes were tested on *S. epidermidis* [113]. Using FIB-SEM and CLFM, recalcitrance toward *Staphylococcus epidermidis* biofilm formation by the nanostructured titanium surfaces was demonstrated.

## 8. Conclusions

The ultrastructural characterization of a biofilm can be carried out by different microscopy methods, however, SEM methods provide the most detailed images at the highest magnifications. Each protocol has its advantages and disadvantages. When high-resolution images, reflecting the actual biofilm ultrastructure, are needed, it is not mandatory the conventional SEM, but an innovative protocol (OsO_4_-RR-TA-IL) is suggested. Using this protocol, the biofilm extracellular matrix becomes conductive and resistant under the electron beam, allowing subsurface structure characterization and observation of bacterial cells as if they were embedded in the matrix. Avoiding dehydration and drying, Eps showed off its actual three-dimensional structure. The combination of innovative SEM protocols with 3-D image analysis software allows for a quantitative evaluation of the 3-D ultrastructure biofilm matrix. In this way, a morphological evaluation takes on the same value as other analytical methods and can be used to compare differences between several anti-biofilm treatments.

## Figures and Tables

**Table 1:** Most widely used non-electron microscopic techniques for biofilm study.

	Light Microscopy	CLSM	AFM
Pros	Simple protocols Cheap and easy to perform Large investigation area	Allows single cell visualization and 3-D imaging	Nondestructive technique that works under physiological-like conditions, allowing living biofilms qualitative and quantitative assessment with few treatments, sample 3-D structure reconstruction at nanometer scale.
Cons	Low resolution and magnification power, need for sample staining, gross morphological differentiation, finest details not visible	Use of fluorophores, limited number of reporter molecules, intrinsic biofilm fluorescence can interfere with probes fluorescence	Small scan area (max 150 × 150 µm), no image of bacterial cells sidewalls, possible surface damage during imaging due to tip interactions.
Applications	Visualization of biofilm formation and quantitative assessment of its biomass	Assessment of biofilm structural parameters, Biofilm 3D structure, identification and localization of living and death cells	Quantitative biofilm analysis, determination of adhesion forces, biofilm topography, in situ imaging.

**Table 2:** *S. mutans* processing procedures and operating conditions from [50].

	Protocols		
Steps	Conventional SEM	VP-SEM	OsO_4_-RR-TA-IL
**Fixation**	Glutaraldehyde 2.5% in PB 0.1 M pH 7.4 at least 48 h		
**Washing**	10 min × 2 times in PB 0.1 M pH 7.4		
**Post-fixation**	OsO_4_ 2% 1 h	OsO_4_ 2% in 1 h	OsO_4_ 2% + RR 0.2% 1:1 solution, 1 h
**Washing**	10 min × 2 times in dH_2_0		
**Impregnation**	None	None	Tannic Acid 1% in d H_2_0 30 min
**Washing**	-	-	10 min × 2 times in dH_2_0
**Dehydration**	Ascending ethanol series	None	None
**Drying**	Ascending HMDS ^1^ series	None	None
**Pt Sputter coating**	15 mA, 2 min	None	Replaced by IL
**Operating conditions**	15–20 kV, high vacuum	5–10 kV 30 Pa	15–20 kV, high vacuum

^1^ HMDS: Hexamethyldisilazane, HN[Si(CH3)3]2T.

**Table 3:** Parameters for the protocol evaluation (from [50]).

	Protocols		
Parameters	Conventional SEM	VP-SEM	OsO_4_-RR-TA-IL
Procedure time	2 days	1 h and 30 min	2 h and 10 min
Sample loss	Steps produce sample loss of about 60%	about 20%	about 20%
Dehydration and drying	yes	None	None
Pt Sputter coating	yes	None	Replaced by IL
Resistance in vacuum	Excellent, it is possible to observe for hours	Good for 1 h	Excellent, it is possible to observe for hours
Operating conditions	15–20 kV, high vacuum	5–10 kV 30 Pa	15–20 kV, high vacuum
Image magnification	Good up to 40 k	Good up to 10 k	Good up to 30 k
Image quality	Excellent up to 30 k	Good up to 8 k	Excellent up to 30 k

## Data Availability

The data presented in this study are available on request from the corresponding author.
